# Performance of a Mobile Phone App-Based Participatory Syndromic Surveillance System for Acute Febrile Illness and Acute Gastroenteritis in Rural Guatemala

**DOI:** 10.2196/jmir.8041

**Published:** 2017-11-09

**Authors:** Daniel Olson, Molly Lamb, Maria Renee Lopez, Kathryn Colborn, Alejandra Paniagua-Avila, Alma Zacarias, Ricardo Zambrano-Perilla, Sergio Ricardo Rodríguez-Castro, Celia Cordon-Rosales, Edwin Jose Asturias

**Affiliations:** ^1^ Section of Pediatric Infectious Diseases University of Colorado School of Medicine Aurora, CO United States; ^2^ Center for Global Health Colorado School of Public Health Aurora, CO United States; ^3^ Children's Hospital of Colorado Aurora, CO United States; ^4^ Department of Epidemiology Colorado School of Public Health Aurora, CO United States; ^5^ Centro de Estudios en Salud, Universidad del Valle de Guatemala Guatemala City Guatemala; ^6^ Department of Biostatistics and Informatics Colorado School of Public Health Aurora, CO United States; ^7^ Division of Health Care Policy and Research University of Colorado School of Medicine Aurora, CO United States; ^8^ Fundacion para la Salud Integral de los Guatemaltecos, Center for Human Development Coatepeque Guatemala; ^9^ Center for Public Health Initiatives Perelman School of Medicine Philadelphia, PA United States; ^10^ Integra IT Bogota Colombia

**Keywords:** mobile phone, app, participatory, syndromic surveillance, norovirus, dengue, acute febrile illness, diarrhea, Guatemala

## Abstract

**Background:**

With their increasing availability in resource-limited settings, mobile phones may provide an important tool for participatory syndromic surveillance, in which users provide symptom data directly into a centralized database.

**Objective:**

We studied the performance of a mobile phone app-based participatory syndromic surveillance system for collecting syndromic data (acute febrile illness and acute gastroenteritis) to detect dengue virus and norovirus on a cohort of children living in a low-resource and rural area of Guatemala.

**Methods:**

Randomized households were provided with a mobile phone and asked to submit weekly reports using a symptom diary app (Vigilant-e). Participants reporting acute febrile illness or acute gastroenteritis answered additional questions using a decision-tree algorithm and were subsequently visited at home by a study nurse who performed a second interview and collected samples for dengue virus if confirmed acute febrile illness and norovirus if acute gastroenteritis. We analyzed risk factors associated with decreased self-reporting of syndromic data using the Vigilant-e app and evaluated strategies to improve self-reporting. We also assessed agreement between self-report and nurse-collected data obtained during home visits.

**Results:**

From April 2015 to June 2016, 469 children in 207 households provided 471 person-years of observation. Mean weekly symptom reporting rate was 78% (range 58%-89%). Households with a poor (<70%) weekly reporting rate using the Vigilant-e app during the first 25 weeks of observation (n=57) had a greater number of children (mean 2.8, SD 1.5 vs mean 2.5, SD 1.3; risk ratio [RR] 1.2, 95% CI 1.1-1.4), were less likely to have used mobile phones for text messaging at study enrollment (61%, 35/57 vs 76.7%, 115/150; RR 0.6, 95% CI 0.4-0.9), and were less likely to access care at the local public clinic (35%, 20/57 vs 67.3%, 101/150; RR 0.4, 95% CI 0.2-0.6). Parents of female enrolled participants were more likely to have low response rate (57.1%, 84/147 vs 43.8%, 141/322; RR 1.4, 95% CI 1.1-1.9). Several external factors (cellular tower collapse, contentious elections) were associated with periods of decreased reporting. Poor response rate (<70%) was associated with lower case reporting of acute gastroenteritis, norovirus-associated acute gastroenteritis, acute febrile illness, and dengue virus-associated acute febrile illness (*P*<.001). Parent-reported syndromic data on the Vigilant-e app demonstrated agreement with nurse-collected data for fever (kappa=.57, *P*<.001), vomiting (kappa=.63, *P*<.001), and diarrhea (kappa=.61, *P*<.001), with decreased agreement as the time interval between parental report and nurse home visit increased (<1 day: kappa=.65-.70; ≥2 days: kappa=.08-.29).

**Conclusions:**

In a resource-limited area of rural Guatemala, a mobile phone app-based participatory syndromic surveillance system demonstrated a high reporting rate and good agreement between parental reported data and nurse-reported data during home visits. Several household-level and external factors were associated with decreased syndromic reporting. Poor reporting rate was associated with decreased syndromic and pathogen-specific case ascertainment.

## Introduction

Given numerous endemic and emerging infectious diseases, there is a need for improved surveillance capacity in low- and middle-income countries (LMICs). Mobile health (mHealth) systems, which take advantage of the increasing availability of mobile communication technology, offer a potential cost-effective tool to improve surveillance capacity in LMICs by obtaining real-time reporting directly from users.

Health care workers (HCWs) have successfully used mHealth platforms in LMICs for disease surveillance, transmission mapping of pathogens, and decision support [1-5]. However, these systems still rely mostly on passive voluntary reporting at health care facilities, which leads to significant underreporting [6,7]. In addition, the World Health Organization estimates a current shortage of more than 7 million HCWs and this shortage is expected to rise to 12.9 million HCWs by 2035, limiting the capacity of HCWs to report disease [8].

An alternative approach to centralized surveillance is to “task shift” data collection from HCWs and health centers to the health care consumer. Participatory syndromic surveillance systems use mHealth tools for community members to directly self-report symptoms indicative of a particular disease [9,10]. These systems, which have traditionally relied on email- or Internet-based reporting, are now transitioning to short message service (SMS) text messages and, more recently, mobile phone app platforms [9,11]. Participatory syndromic surveillance has been used in developed countries for influenza and syndromic (ie, acute respiratory illness) surveillance in the last decade, but their use in LMICs has been limited, especially in combination with diagnostic testing [9,11-15]. Similar to most LMICs, Guatemala has limited experience with participatory syndromic surveillance and relies almost entirely on passive, centralized surveillance systems to estimate disease burden, including for important emerging pathogens such as dengue virus and norovirus, two of the most common causes of fever and diarrhea, respectively [16-19].

To better understand the utility of participatory syndromic surveillance for emerging disease surveillance in LMICs, we studied the performance and acceptability of a mobile phone app-based participatory syndromic surveillance system in detecting acute febrile illness and acute gastroenteritis among a randomized cohort of children in a low-resource region of Guatemala with limited access to Internet and telecommunication.

## Methods

### Study Setting and Population

The study was conducted in 25 communities within a 200 km^2^ catchment area along the coastal lowlands of southwest Guatemala. The populations living in these communities suffer from high levels of food insecurity, poverty, low access to health care, and high levels of diarrheal and respiratory disease [20]. Households with children aged 6 weeks to 17 years were eligible for study inclusion. All children within a consenting household were offered enrollment, and the household was included if at least one child participated. The household was excluded if the parent was unable to demonstrate proficiency in submitting the weekly mobile phone-based symptom diary after instruction.

### Mobile Phone App Development and Use

An encrypted Android mobile phone app (Vigilant-e), developed by Integra IT (Bogota, Colombia), was used. The app allows study participants to directly enter and report symptoms or events using simplified question algorithms and decision-tree logic (see Multimedia Appendix 1 for Vigilant-e app screenshots). Once submitted, encrypted participant data are automatically uploaded using cellular data networks into a secure central database and removed from the mobile phone. Data submitted into the database are available in real time and automated alerts can be generated and sent electronically to study personnel, identifying participants meeting predefined case definitions (acute febrile illness or acute gastroenteritis) or Integrated Management of Childhood Illness (IMCI) warning signs [21].

The Vigilant-e app was configured with the participation of the study investigators, the study nurses, community members, and the Integra IT team. Given low education rates and mobile phone use in the region, the user interface was simplified to a minimum number of questions with simple language and visual aids when possible. Study personnel were trained and the app was field-tested to determine if mobile data coverage was acceptable. Although Internet coverage was variable, the Vigilant-e app was able to store data locally until data coverage was acquired, at which point the data were automatically uploaded into the study database. To avoid unnecessary consumption of available data from participants, all apps on their mobile phones were blocked except for Vigilant-e and WhatsApp as well as phone and text messaging apps, so participants could communicate with study nurses when needed.

### Case Definitions

Case definitions were created prior to study initiation. Acute gastroenteritis was defined as self-reported vomiting or diarrhea for at least 3 days or both for 1 day or more in the preceding week. Norovirus-associated acute gastroenteritis was defined as acute gastroenteritis with concurrent positive norovirus reverse transcription polymerase chain reaction (RT-PCR) testing at the time of sampling. Acute febrile illness was defined as self-reported fever for at least 2 days within the preceding week. Dengue fever was defined as acute febrile illness with positive dengue virus RT-PCR test or anti-DENV IgM serologic test at the time of sampling. If a child reported acute gastroenteritis or acute febrile illness for more than 2 weeks in a row, only the clinical data and sample from the first week were included.

### Surveillance System

All households were screened and enrolled using a two-stage cluster sampling strategy adapted from the World Health Organization lot quality assurance method, in which 30 clusters of 7 households were enrolled at random within the study catchment area, as previously described [22-25]. Demographic, geographic information system (GIS), exposure, and clinical data were collected at enrollment, including a survey on the presence and use of mobile phones in the household. All participating households were provided a study-specific mobile phone (Huawei Y330) with the Vigilant-e symptom diary app installed and were asked to complete a symptom report (fever, vomiting, or diarrhea) each week on each participating child. For positive reports of fever, vomiting, or diarrhea, the study nurses phoned the participants to confirm symptoms and ask additional clinical questions. If no weekly report was received from a household in a given week, a study nurse phoned the parent to remind them of the submission requirement. Any participant who met the case definition for acute febrile illness or acute gastroenteritis on their weekly report was visited at their home by a study nurse, with a goal interval time between report and home visit of less than 48 hours. During this visit, study nurses would repeat the same questions asked on the symptom diary app and would obtain a stool or blood sample from participants reporting acute gastroenteritis and acute febrile illness, respectively. Any participant with IMCI danger signs was referred to a medical facility; study physicians supervised all nurses.

Beginning on October 2, 2015, if a participant did not submit the symptom diary in a given week, study nurses were allowed to phone the household and manually enter weekly symptom diary data into the study database instead of relying entirely on the Vigilant-e app. Beginning in early April 2016, all participating households were visited by a study nurse, any malfunctioning phones were replaced, and participants were reminded to submit weekly symptom data using the app. Prospective surveillance using the weekly symptom diary was continued through June 2016, at which point a final closeout visit was performed.

### Laboratory Testing

Serum samples from participants with acute febrile illness during prospective follow-up were tested for dengue virus by RT-PCR using the Centers for Disease Control and Prevention’s dengue virus assay (DENV-1-4) and IgM anti-DENV IgM enzyme-linked immunosorbent assay (ELISA, InBios Inc, Seattle, WA, USA). Stool specimens were collected using Copan FLOQSwabs (Brescia, Italy) either by rectal swab or on fresh (<2 hours old) stool sample and eluted in eNAT transport solution (Copan, Brescia, Italy) before testing, with both collection techniques previously demonstrating similar molecular viral yield [26,27]. Samples were stored on-site at –20°C and shipped on dry ice to Universidad del Valle de Guatemala (UVG) where diagnostic testing was performed as previously described [25,28].

### Statistical Analysis

Demographic variables were compared between the households with 70% or greater response rate and those with less than 70% response rate using a generalized linear model with at binomial response distribution and a log link function. The 70% cutoff was chosen based on response rates observed in previous participatory syndromic surveillance studies in non-LMICs [12,13,29,30]. Agreement of symptoms reported on the Vigilant-e app versus those reported by study nurses at the home visits were calculated using Kendall tau for continuous variables and kappa statistic for categorical variables. SAS v 9.4 (Cary, NC, USA) was used for all data analysis. Mapping of households by response rate was performed using the “sp” spatial package in R and Google satellite images [31].

### Ethical Oversight

The study was approved by the Colorado Multiple Institutional Review Board, the UVG Institutional Review Board, and the Guatemala Ministry of Health National Ethics Committee. The local Southwest Trifinio Community Advisory Board for Research agreed to the study.

## Results

**Figure 1:**
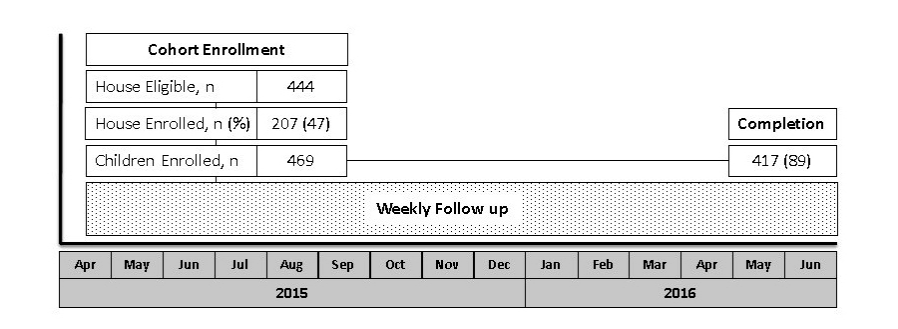
Study design and CONSORT diagram of study recruitment, enrollment, and completion. The participatory syndromic surveillance (PSS) cohort enrolled children from April to September 2015, followed by prospective observation with the weekly symptom diary for acute gastroenteritis and acute febrile illness episodes (dotted box) through June 2016.

The study enrolled 207 of 444 (46.6%) eligible households from April to September 2015, which included 469 children (Figure 1). The most commonly cited reasons for declining study participation (n=73) included lack of perceived benefit to the child (21/73, 29%), discomfort with rectal specimen collection (16/73, 22%), and not wanting responsibility for the mobile phone (12/73, 16%). Enrolled households were 97.1% (201/207) nonindigenous with a mean of 5.0 (SD 1.8) people living in the household, including mean 2.6 (SD 1.4) children younger than 18 years with a mean age of 7.3 (SD 4.7) years (Table 1). Household density was semirural with a mean of 9.5 (SD 8.6) households per 300 m^2^.The primary caregiver (and study mobile phone user in most cases) was literate in 88.4% (183/207) of households; 24.6% (51/207) of fathers and 18.4% (38/207) of mothers had completed secondary education.

Cellular phone ownership among households at enrollment varied with 12.1% (25/207) reporting no phone in the household at study initiation and 39.6% (82/207) reporting the use of a mobile phone. Of those households with cellular phones, 72.5% (150/207) used them for text messaging and 37.9% (69/182) for Internet access, of which 19.7% (13/66) accessed the Internet at least daily.

**Figure 2:**
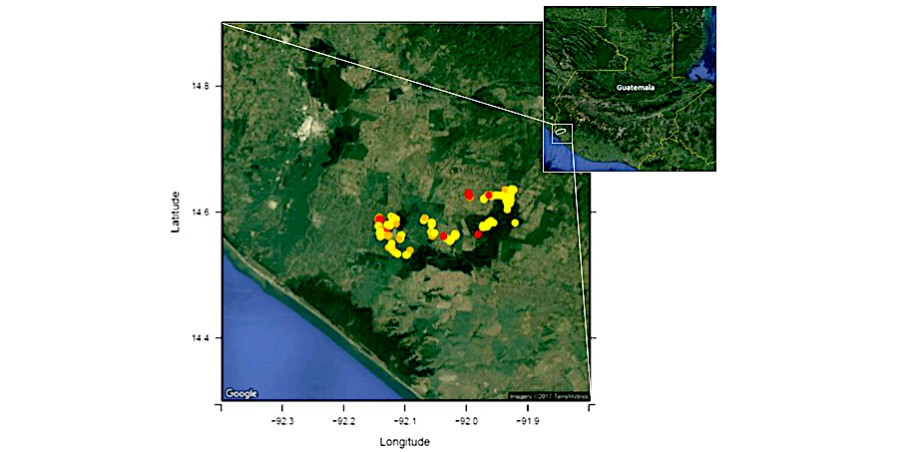
Map showing clusters of participants with high (>70%; yellow circle) symptom diary app response rate versus moderate (40%-70%; orange circle) and low (<40%; red circle) response rates, in the Southwest Trifinio Region of Guatemala during the first 25 weeks of surveillance prior to allowing study nurses to manually enter syndromic data (April-October 2015).

From April 2015 to June 2016, enrolled participants completed 471 person-years of prospective observation with a mean weekly symptom reporting rate of 78% (range 58%-89%) among enrolled children. During the first 25 weeks of observation, study participants with a low (<70%) weekly parental symptom reporting rate using the Vigilant-e app were more likely to be female than participants with a high (≥70%) weekly reporting rate (57% vs 44%, risk ratio [RR] 1.4, 95% CI 1.1-1.9). Households with a low (<70%) weekly symptom reporting rate (n=57) were more likely to have a greater number of children (mean 2.8, SD 1.5 vs mean 2.5, SD 1.3; RR 1.2, 95% CI 1.1-1.4), were less likely to have used mobile phones for text messaging at study enrollment (61% vs 77%, RR 0.6, 95% CI 0.4-0.9), and were less likely to access health care at the local public clinic (35% vs 67%, RR 0.4, 95% CI 0.2-0.6) than households with a ≥70% reporting rate (Table 1). Household cluster density did not differ between households with low versus high reporting rates and there was no significant difference in reporting rates based on geolocation (Figure 2). Households with low weekly reporting rates were significantly less likely to report acute gastroenteritis, norovirus-associated acute gastroenteritis, acute febrile illness, and dengue virus-associated acute febrile illness than households with high weekly reporting *(P<*.01, Table 1). The most commonly cited reasons for not submitting weekly reports were poor data signal, discomfort with using the mobile phone, data consumption, stolen or lost phone, and forgetting to send the report.

**Figure 3:**
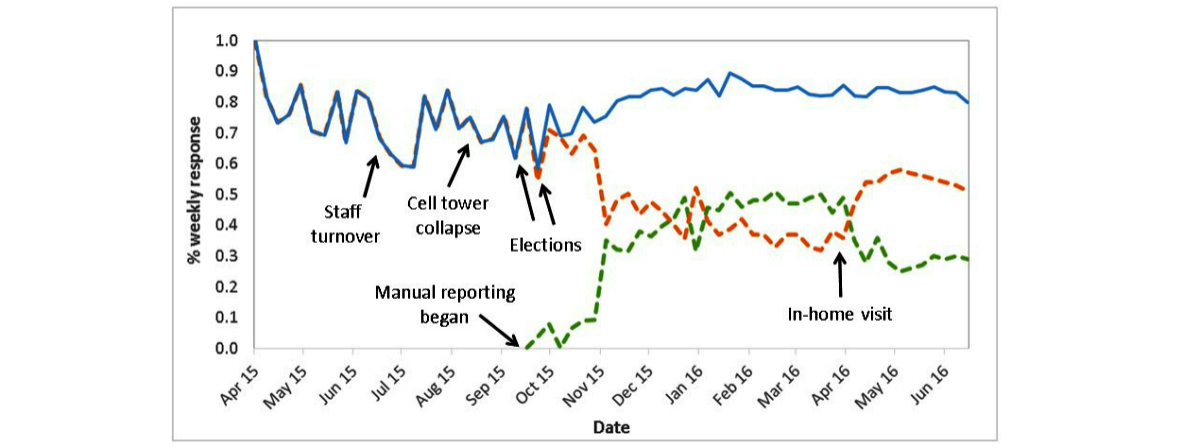
Weekly syndromic reporting rate for acute febrile illness and acute gastroenteritis, April 2015-June 2016. Weekly syndromic reporting rate of participants using the Vigilant-e symptom diary mobile phone app (orange), manually entered data from nurse phone call (green), and combined mobile phone and manual data entry (blue). Several factors were associated with periods of decreased reporting, including a time period of high staff turnover (June-July 2015), a cellular tower collapse (August 2015), and primary and run-off presidential elections (October 2015). On October 2, 2015, study nurses were allowed to manually enter participant data if there was no response. In April 2016, study nurses performed an in-home visit to participating households to repair or replace malfunctioning phones and remind participants to use the Vigilant-e app for reporting when possible.

Several external factors disrupted the weekly symptom reporting by study participants, including a period of staff turnover, the collapse of a local cellular tower, and contentious primary and secondary national elections (Figure 3). After October 2015, when nurses were allowed to manually enter symptom data directly into the study database for individuals failing to submit symptom data using the Vigilant-e app in a given week, mean overall weekly reporting increased from 73% to 82% over an 8-week period *(P<*.001) (Figure 3). However, during the same timeframe, there was an associated decrease of parents self-reporting symptom data using the Vigilant-e app from 100% to 60% (*P*<.001). After April 2016, when nurses performed the intervention to improve symptom reporting using the Vigilant-e app, the proportion of households self-entering symptom data using the Vigilant-e app increased from 36% preintervention to 54% 4 weeks postintervention (*P*=.046), and the overall mean weekly reporting rate (self-report and nurse report) remained unchanged at 83% (*P*=.93).

When comparing symptom reporting using the Vigilant-e app to nurse-recorded reporting at the home visit (conducted within 48 hours), there was strong agreement between the Vigilant-e app and the home visit for all symptoms except bleeding, which was rarely reported (Table 2). The average time interval between an acute febrile illness or acute gastroenteritis report and a nurse home visit was mean 1.2 (SD 1.7) days; 69.9% (79/113) of participants were visited within 1 day of a report and 86.7% (98/113) were visited within 2 days of a report. The greater the time between the Vigilant-e app symptom report and the nurse home visit, the greater the disagreement in the symptoms (fever, vomiting, and diarrhea) between reporters (Table 3). Overall, at the time of study completion, there was a high satisfaction rate with study participation with 178 (98.8%) of households reporting that participating in the study was beneficial to them personally and 174 (96.6%) reporting that participation benefited the community.

**Table 1:** Study participant and household characteristics and risk factors associated with low symptom diary app response rate (&lt;70%), April to September 2015.

Characteristics			Overall			Response rate ≥70%		Response rate <70%			RR (CI)^b^ (ref ≥70%)		*P*	
**Child characteristics**^a^														
	Children enrolled, n			469			322		147					
	Age (years), mean (SD)			7.3 (4.7)			7.1 (4.8)		7.5 (4.4)			1.0 (1.0-1.04)		.44
	Gender (female), n (%)			225 (47.9)			141 (43.8)		84 (57.1)			1.4 (1.1-1.9)		.008
	Child vaccinated (rotavirus), n (%)			250 (53.3)			165 (51.2)		85 (57.8)			1.2 (0.9-1.7)		.13
	Child attends school (if age ≥6 years), n (%)			217 (85.1)			143 (84.1)		74 (87.1)			0.8 (0.5-1.4)		.55
**Household characteristics**														
	Households enrolled, n			207			150		57					
	Individuals in house, mean (SD)			5.0 (1.8)			4.9 (1.7)		5.2 (2.0)			1.1 (0.9-1.2)		.27
	Children enrolled per household, mean (SD)			2.6 (1.4)			2.5 (1.3)		2.8 (1.5)			1.2 (1.1-1.4)		.004
	Children aged ≤5 years enrolled per household, mean (SD)			1.0 (0.8)			1.0 (0.8)		1.0 (0.8)			1.0 (0.8-1.4)		.81
	Household cluster density, mean (SD)			9.5 (8.6)			9.9 (3.4)		8.1 (8.0)			0.8 (0.6-1.04)		.09
	Primary caregiver literacy, n (%)			183 (88.4)			131 (87.3)		52 (91)			1.3 (0.6-3.0)		.52
	Father’s education ≥secondary, n (%)			51 (24.6)			34 (22.7)		17 (30)			1.3 (0.8-2.1)		.28
	Mother’s education ≥secondary, n (%)			38 (18.4)			32 (21.3)		6 (11)			0.5 (0.2-1.1)		.10
	Health care at public clinic, n (%)			121 (58.5)			101 (67.3)		20 (35)			0.4 (0.2-0.6)		<.001
	Duration at current house (years), mean (SD)			8.1 (3.4)			8.04 (3.4)		8.34 (8.8)			1.0 (0.9-1.1)		.63
**Cellular phone usage**														
	Cellular phones per household, mean (SD)			1.4 (1.1)			1.4 (1.0)		1.4 (1.1)			1.0 (0.8-1.2)		.95
	**Most advanced phone in household, n (%)**													
		No phone			25 (12.1)		16 (10.7)			9 (16)		Ref		
		No mobile phone			99 (47.8)		73 (48.7)			26 (46)		0.7 (0.4-1.4)		.32
		Mobile phone			82 (39.6)		60 (40.0)			22 (39)		0.7 (0.4-1.4)		.36
	Phones used for text messaging, n (%)^c^			150 (72.5)			115 (76.7)		35 (61)			0.6 (0.4-0.9)		.02
	Uses a phone with Internet, n (%)^c^			69 (37.7)			56 (42.1)		13 (27)			0.6 (0.4-1.1)		.10
	**Internet access frequency**^d^, **n (%)**													
		≤Weekly			53 (80.3)		45 (81.8)			8 (73)		Ref		
		≥Daily			13 (19.7)		10 (17.8)			3 (27)		1.5 (0.5-5.0)		.48
**Syndromic reporting (Apr 2015-Jun 2016)**^e^														
	**Acute gastroenteritis, n (%)**			100			92 (0.7)		8 (0.3)			0.2 (0.1-0.4)		<.001
		Norovirus-associated acute gastroenteritis, n (%)					12 (3.7)			0 (0)		N/C^f^		N/C
	**Acute febrile illness, n (%)**			122			112 (0.9)		10 (0.3)			0.2 (0.1-0.4)		<.001
		Dengue-associated acute febrile illness, n (%)					4 (1.2)			0 (0)		N/C		N/C

^a^We were unable to model the random effects of multiple children per household due to relatively low numbers of children per household.

^b^Risk ratios (RR) and 95% confidence intervals were calculated using univariate generalized linear models, with dichotomous response rate in the first 25 weeks of surveillance (≥70% vs <70%) as the outcome of interest.

^c^12% of households are missing these variables.

^d^68% of all households are missing this variable because they said they did not use a phone with Internet access in the previous question.

^e^The response rates reflect the first 25 weeks of surveillance, despite the longer syndromic reporting period (April 2015-June 2016).

^f^N/C: not calculated

**Table 2:** Agreement of symptom reporting among study participants between mobile phone symptom diary app and nurse home visit, April 2015 to June 2016.

Symptoms^a^	Symptom diary app (n=113)	Nurse home visit (n=113)	Kappa or Kendall tau^b^	*P*^b^
Fever, n (%)	62 (56.9)	79 (69.9)	.57	<.001
Fever duration (days), mean (SD)	2.9 (1.3)	3.0 (1.8)	.46	<.001
Rash, n (%)	15 (24.2)	16 (20.3)	.59	<.001
Pain, n (%)	38 (61.3)	45 (57.0)	.55	<.001
Nausea, n (%)	29 (46.8)	32 (40.5)	.48	<.001
Bleeding, n (%)	3 (4.8)	1 (1.3)	–.02	.82
Vomiting, n (%)	62 (57.4)	29 (25.7)	.63	<.001
Duration (days), mean (SD)	2.5 (2.0)	1.9 (0.9)	.69	<.001
Maximum emesis/day, mean (SD)	4.5 (2.8)	3.6 (1.7)	.56	.002
Diarrhea, n (%)	33 (30.3)	70 (62.0)	.61	<.001
Diarrhea duration (days), mean (SD)	3.2 (1.8)	3.4 (1.8)	.78	<.001
Maximum stools/day, mean (SD)	4.7 (2.1)	5.1 (2.2)	.29	.006

^a^Participants were asked additional symptom questions if they responded that “yes” their child had fever, diarrhea, or vomiting on the app or the nurse phone call. Nurses also asked the same questions using the same screening technique at the home visit (along with many more detailed questions). Symptoms included any reported symptom, regardless of duration.

^b^Kappa statistic for categorical variables and Kendall tau for continuous variables.

**Table 3:** Agreement between self-reported symptoms using the Vigilant-e app and study nurse-collected symptoms at home visit.

Days between app report and home visit^a^	n	Fever			Vomiting			Diarrhea	
		Kappa	*P*	Kappa		*P*	Kappa		*P*
<1	79	.70	<.001	.66		<.001	.65		<.001
1	19	.51	.03	.68		.002	.76		.002
≥2	15	.08	.71	.28		.29	.13		.64

^a^As the time interval increased between self-reported symptoms (Vigilant-e app) and nurse-collected symptoms (home visit), agreement between these reporting mechanisms decreased (kappa coefficient). If nurse-collected symptoms occurred within 1 day of self-report, kappa agreement was .65-.70.

## Discussion

In a resource-limited region of Guatemala with low literacy rates, we implemented a mobile phone-based participatory syndromic surveillance system with a high weekly response rate and a high rate of agreement between mobile phone parental reporting and nurse home visit reporting. We identified individual and community factors that led to decreased reporting, including female sex of the study participant, a greater number of children in the home, less prior experience with SMS text messaging, and lower utilization of local public health clinics. During our surveillance period, several external factors were associated with decreased reporting, including a cellular tower collapse and national elections, which highlight novel problems in conducting mobile phone-based surveillance. In addition, we demonstrated how contact with study participants, either with phone calls or home visits, may influence self-reporting. Finally, in this region of high norovirus and dengue virus burden [17,25], we found an association between lower rate of weekly self-report and fewer detected episodes meeting syndromic (acute febrile illness and acute gastroenteritis) and pathogen-specific (dengue virus and norovirus) case definitions.

Conducting prospective and accurate infectious disease surveillance in resource-limited settings is difficult for many reasons, including a lack of trained personnel, infrastructure, and diagnostic testing. Prospective surveillance studies have traditionally used weekly home visits or phone calls to collect syndromic data, followed by diagnostic testing in individuals meeting predefined case definitions, although these systems require significant resources and personnel [19,32]. In our study, we task-shifted syndromic data collection directly to community members by training them to use a mobile phone-based symptom diary app. Although study nurses were still required to phone households that did not submit a report in a given week, and we showed that regular contact between study nurses and participants improved reporting, the vast majority of syndromic reporting was entered directly by study participants into an electronic database. The parent-reported data were also accurate when compared to data collected by study nurses, especially when the nurse home visit occurred the same day as the submitted report. Although local syndromic data are unavailable, our estimated acute febrile illness incidence (18.7 per 100 person-years) was similar to that in multiple Latin American countries using a weekly home visit system (26.7 per 100 person-years) [19]. Our estimated acute gastroenteritis incidence (21.0 per 100 person-years) was lower than estimates reported elsewhere (37-400 episodes per 100 person-years), although these other studies generally included less strict case definitions (1 day of symptoms instead of 3 days), younger populations (<5 years instead of <18 years), and still demonstrated a wide variability in acute gastroenteritis incidence [18,33].

We identified several practical lessons in performing mobile phone-based participatory syndromic surveillance in this resource-limited area. Although mobile phones are increasingly integrating into these communities, the telecommunication infrastructure is susceptible to interference, as demonstrated by decreased reporting following a cellular tower collapse. In addition, other external factors, including a period of high staff turnover that delayed reminder phone calls and widespread protests that impacted both participants and study personnel, were associated with periods of decreased self-reporting. Users were provided with limited data use per month and phones were locked to prevent use of non-study apps, but participants still found ways to circumvent this process. We found that regular communication between study personnel and participants led to improved reporting, but this required more personnel. Allowing study nurses to manually enter participant data into the database, instead of the participants, led to improved reporting overall but was associated with decreased self-reporting, somewhat undermining the participatory syndromic surveillance system. Prior to this intervention, the reporting rate (74%) was consistent with participatory syndromic surveillance studies in non-LMICs [12,13,29,30], and depending on the needs of the surveillance system (eg, vaccine effectiveness, outbreak response), may be sufficient. Further studies are needed to better define the role of participatory syndromic surveillance in these specific settings, and to optimize the interaction between participant and study personnel.

As mobile phones and data networks become increasingly integrated into resource-limited regions of the world, mobile phone-based participatory syndromic surveillance will likely become a more powerful tool to collect population-level syndromic data. Although we provided mobile phones to participants in our study, an important future step will be to allow users to download a symptom diary app onto their own mobile phones and encouraging syndromic self-reporting by providing some small incentive, such as prepaid airtime. This strategy, although still requiring personnel to maintain communication with participants and collect samples when needed, would allow a significant scale-up of the surveillance platform. And although syndromic data would be collected for all individuals, limiting diagnostic testing to a random or higher risk (meeting a more specific case definition) subset of participants would significantly reduce costs. This type of system could also be used for specific populations, such as in screening pregnant women for Zika virus, or during outbreaks of emerging pathogens such as Ebola. Because access to mobile phones is not evenly distributed within a population, it will be important for these types of surveillance programs to find strategies to ensure population-level representativeness.

The study had several strengths and limitations. We chose to perform the study in one of the most resource-limited regions of Guatemala where 70% of the population reports food insecurity and where 60% of households do not own a mobile phone [20]; success in this setting supports the likelihood of replicating this system in other areas with equal or greater resources. Individuals who owned mobile phones at baseline had attained a higher level of education compared to those that did not own mobile phones (*P*<.001), and they presumably had higher socioeconomic status, so we provided a study mobile phone to all enrolled households to limit bias in our study population. Nevertheless, many eligible households (>50%) declined participation, including 16% that did not want responsibility for the mobile phone, which may have led to a selection bias. We were unable to determine whether the decreased syndromic reporting in the low (<70%) response group was due to actual decreased disease incidence or due to decreased reporting. The representativeness of the system may be biased toward high (≥70%) response households, which is an important consideration for future studies. Sample collection within our prospective syndromic surveillance system decreased over time secondary to parental refusal [25], and although syndromic reporting was maintained at a high level throughout the study, it is possible that households were not reporting symptoms to avoid diagnostic testing (venipuncture or rectal/stool swab). This possibility could have been evaluated by sending study nurses at random to households that reported no symptoms in a given week and comparing their data to the mobile phone self-report. Households did report that the study, including the nurse home visits, benefited their families (98.8%) and the community (96.6%) overall.

In summary, we successfully implemented a mobile phone-based participatory syndromic surveillance system in a resource-limited region of Guatemala and identified several factors that positively or negatively impacted self-reporting. Self-reporting using a symptom diary mobile phone app (Vigilant-e) was accurate when compared to study nurse-collected data during a home visit. Future studies should evaluate mobile phone-based participatory syndromic surveillance for specific high-risk populations and pathogens at other sites, and should scale-up syndromic self-reporting with diagnostic testing performed only within a randomized or select subset of responders.

## Appendix

Multimedia Appendix 1 Sample screenshots from the Vigilant-e application (Integra IT, Bogota, Colombia) for weekly participatory syndromic surveillance in the Trifinio Region of Guatemala. Each week, subjects would select if any of the children enrolled from their household had fever or rash (Panel A). If the parent or guardian reports symptoms (red box), a decision-tree logic would then ask them to report symptoms for each child (Panel B). If they selected the child and then reported fever, vomiting, or diarrhea (not shown), another screen would appear asking additional syndromic questions (Panel C).

## Abbreviations

ELISA: enzyme-linked immunosorbent assay
HCW: health care workers
IMCI: Integrated Management of Childhood Illness
LMIC: low- and middle-income countries
N/C: not calculated
RR: risk ratio
RT-PCR: reverse transcription polymerase chain reaction
