# Supplementary figures and images for: Performance of a Mobile Phone App-Based Participatory Syndromic Surveillance System for Acute Febrile Illness and Acute Gastroenteritis in Rural Guatemala
Source: J Med Internet Res. 2017 Nov 9;19(11):e368. doi: 10.2196/jmir.8041 (PMC5701088; doi:10.2196/jmir.8041)

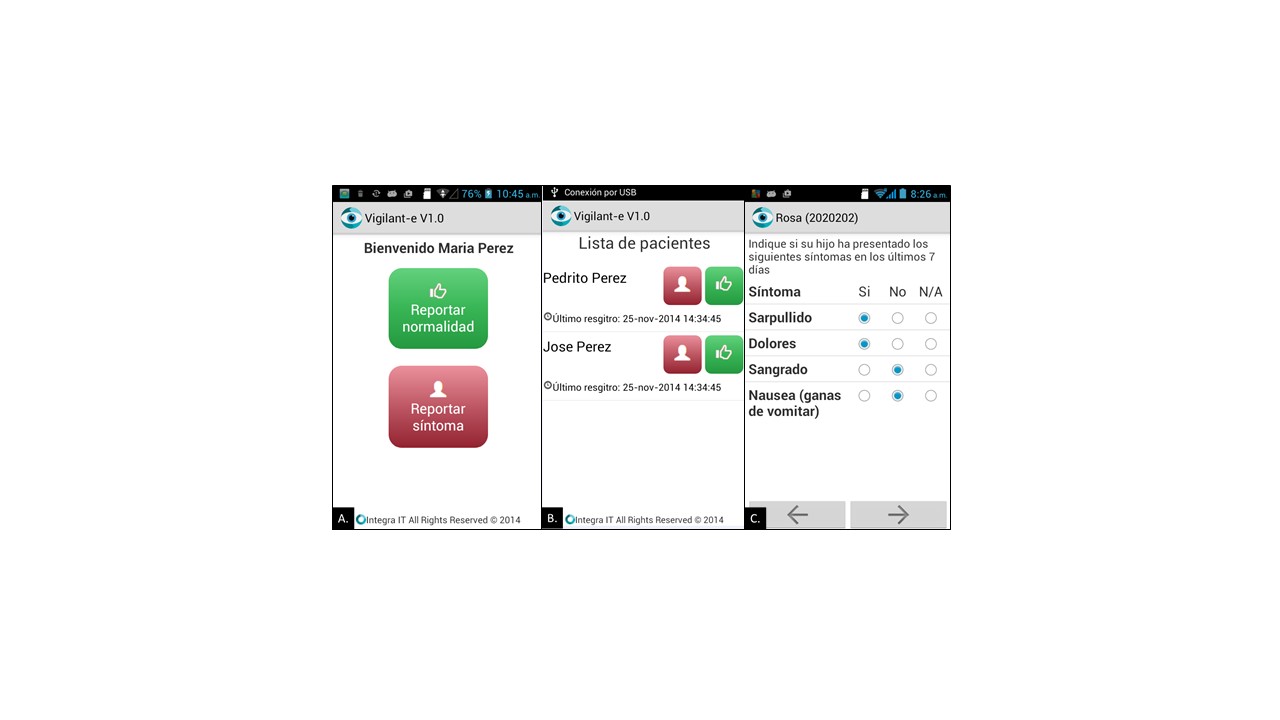

Supplement: Multimedia Appendix 1 [file jmir_v19i11e368_app1.JPG]
